# Intraspecific variation among Tetranychid mites for ability to detoxify and to induce plant defenses

**DOI:** 10.1038/srep43200

**Published:** 2017-02-27

**Authors:** Rika Ozawa, Hiroki Endo, Mei Iijima, Koichi Sugimoto, Junji Takabayashi, Tetsuo Gotoh, Gen-ichiro Arimura

**Affiliations:** 1 Center for Ecological Research, Kyoto University, Otsu 520-2113, Japan; 2 Department of Biological Science & Technology, Faculty of Industrial Science & Technology, Tokyo University of Science, Tokyo 125-8585, Japan; 3 Laboratory of Applied Entomology and Zoology, Faculty of Agriculture, Ibaraki University, Ibaraki 300-0393, Japan

## Abstract

Two genotypes coexist among Kanzawa spider mites, one of which causes red scars and the other of which causes white scars on leaves, and they elicit different defense responses in host plants. Based on RNA-Seq analysis, we revealed here that the expression levels of genes involved in the detoxification system were higher in Red strains than White strains. The corresponding enzyme activities as well as performances for acaricide resistance and host adaptation toward Laminaceae were also higher in Red strains than White strains, indicating that Red strains were superior in trait(s) of the detox system. In subsequent generations of strains that had survived exposure to fenpyroximate, both strains showed similar resistance to this acaricide, as well as similar detoxification activities. The endogenous levels of salicylic acid and jasmonic acid were increased similarly in bean leaves damaged by original Red strains and their subsequent generations that inherited high detox activity. Jasmonic acid levels were increased in leaves damaged by original White strains, but not by their subsequent generations that inherited high detox activity. Together, these data suggest the existence of intraspecific variation - at least within White strains - with respect to their capacity to withstand acaricides and host plant defenses.

Kanzawa spider mite (*Tetranychus kanzawai*, Acari; Tetranychidae) is a pest of herbaceous and woody plants that originated in Southeast Asia, and has now spread to many other countries, including Australia, the Congo Republic, and the USA^1^,^2^. One striking characteristic of this species is that there are two genotypes that make either white scars or red scars on host leaves of several plant taxa, e.g., *Phaseolus* spp., (these genotypes are hereafter referred to as the “White” and “Red” strains)^3^. It was reported that the Red genotype is dominant over the White one, due to differences at a single gene locus^3^. Ecologically, Red strains defoliate more leaves of *P. vulgaris* and disperse from infested *P. vulgaris* leaves earlier than White strains do^3^.

Distinct levels of defense responses are elicited in host plants damaged by either Red or White strain mites: Red strain mites elicit salicylic acid (SA)-induced defense responses more strongly than White strain mites do in leaves of *P. lunatus*, whereas jasmonic acid (JA)-induced defense responses are similarly elicited by both strains^4^. Moreover, *P. lunatus* plants infested with Red strain mites emit higher levels of homoterpenes ((*E*)-4,8-dimethyl-1,3,7-nonatriene, (*Z*)-4,8-dimethyl-1,3,7-nonatriene and (*E,E*)-4,8,12-trimethyl-1,3,7,11-tridecatetraene) in comparison to those infested with White strain mites^4^. Some of these homoterpenes play roles as infochemicals in the attraction of carnivorous natural enemies of spider mites (e.g., *Neoseiulus californicus* and *Phytoseiulus persimilis*)^5^,^6^.

As described above, previous research has revealed an array of indexes for which the performances differ between Red and White strains. However, it is unclear why these two genotypes elicit these differential responses in plants and how this relates to their performance. As the first step to address this issue, we performed RNA-Seq analysis of the two genotypes. The transcriptome analysis, in combination with a series of physiological and ecological assays, provided insights into the intraspecific variation of Kanzawa spider mites.

## Results

### Diverse detoxification abilities among mites sharing the same genotype

Two independent Red strains (R1 and R2) and two independent White strains (W1 and W2) were isolated based on whether red or white scars were produced on damaged host *P. vulgaris* leaves over successive generations (Supplemental Fig. 1). To obtain molecular insight into the different traits of these Red and White strains (see “Introduction”), we carried out transcriptome analysis using RNA-Seq. The sequences of mRNA from adult females were assembled into 34963 (46697) unigenes (contigs) for the R1 strain, 33209 (45567) for the R2 strain, 32191 (54028) for W1, and 32346 (53268) for W2 (Supplemental Table 1). Molecular function distributions from Gene Ontology (GO) analyses using the assembled unigenes + contigs revealed no distinguishable difference of the distribution of expressed genes among strains (Supplemental Fig. 2).

**Figure 1:**
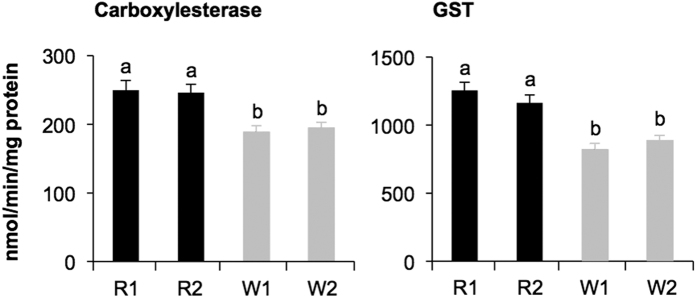
Detoxification enzyme activity in adult females of Red strains (R1 and R2) and White strains (W1 and W2). Data are shown as the mean ± standard errors (*n* = 7–11). Means indicated by different small letters are significantly different, based on a Tukey’s HSD test (α = 0.05) after a one-way ANOVA.

Among the genes (unigenes + contigs) that were expressed at levels differing by at least 2-fold between the two Red strains (R1 and R2) compared to the two White strains (W1 and W2), 790 genes were expressed more highly in the Red strains, while only 63 genes were expressed more highly in the White strains (Supplemental Table 2). Notably, genes involved in the detoxification system (i.e., cytochrome P450 [CYP], glutathione *S*-transferase [GST], carboxylesterase, and ABC transporter) were expressed more highly in the Red strains than in the White strains (33 genes among 493 genes (7%) annotated in the Swiss Prot database or Tetranychus database), whereas none of them were expressed more highly in the White strains (Table 1). This was reflected by the enzyme activity levels of GST and carboxylesterase: the levels of activity of these enzymes were higher in the R1 and R2 strains than in the W1 and W2 strains (Tukey’s HSD test; α = 0.05) (Fig. 1), suggesting that the detoxification activity of Red strains was higher than that of White strains.

Likewise, 10 genes for chaperone proteins, such as heat shock proteins, were expressed more highly in the Red strains than in the White strains (4 genes), whereas no genes for chaperone proteins were expressed more highly in the White strains (Table 2). A suite of genes for regulatory proteins, including transcription factors (7 genes), protein kinases (10 genes), protein phosphatases (7 genes) and ubiquitin ligases, were also expressed more highly in the Red strains (Supplemental Table 3). Expression of selected genes for detoxification enzymes and heat shock proteins were additionally analyzed using reverse transcription-quantitative polymerase chain reaction (RT-qPCR), leading to findings of significant differences between Red and White strains (Tukey’s HSD test; α = 0.05) (Supplemental Fig. 3).

### Host plant adaptation of strains

**Figure 2:**
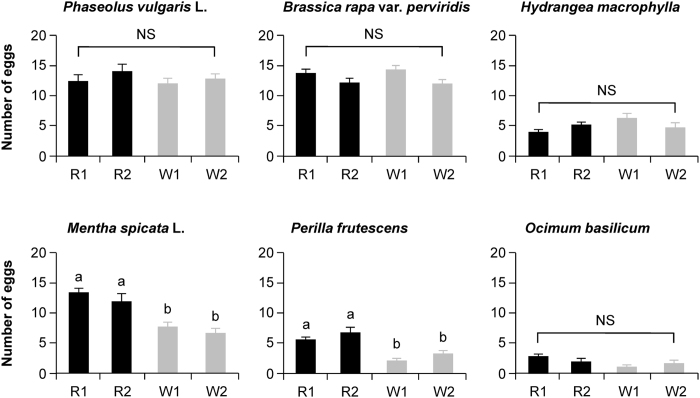
The oviposition rate of Red and White adult females. The number of eggs produced by Red strains (R1 and R2) and White strains (W1 and W2) during 2 days was counted on various host plants. Data are shown as the mean + standard error (*n* = 15–28). Means indicated by different small letters are significantly different, based on a Tukey’s HSD test (α = 0.05) after a one-way ANOVA. NS, not significant.

We hypothesized that, if the Red strains indeed had higher detoxification ability, they would more easily adapt to host plants than the White strains would. We therefore compared the numbers of eggs laid by the R1, R2, W1, and W2 strains on leaf discs of *P. vulgaris* for 48 h, and found no clear differences among the four strains (Tukey’s HSD test; α = 0.05) (Fig. 2). The same held true for the numbers of eggs laid by the two genotypes on three other plant species: (*B. rapa* [Brassicaceae], *H. macrophylla* [Hydrangeaceae] and *O. basilicum* (Laminaceae). In contrast, the two Red strains laid a significantly larger numbers of eggs on leaf discs of two Laminaceae species (*M. spicata* L. and *P. frutescens*) compared to the two White strains (Fig. 2). We note that red and white scars of damage were visually observed only on the host leaves of *P. vulgaris* and *H. macrophylla*, not on the leaves of other host species tested (not shown).

### Acaricide resistance variability

In light of the different detoxification abilities between the Red and White strains, we carried out acaricide resistance assays for them. We used five acaricides that have been broadly used for the control of spider mites: Danitoron^®^ (fenpyroximate: an inhibitor of mitochondrial electron transport at the NADH-coenzyme Q reductase^7^), Kanemaito^®^ (acequinocyl: mitochondria complex III inhibitor^8^), Koromaito^®^ (milbemycin: a neurotoxin, acting as a GABA agonist in the nervous system^8^), Maitokone^®^ (bifenazate: a mitochondrial complex III inhibitor^9^), and Starmite^®^ (cyenopyrafen: a mitochondria complex II inhibitor^10^). When fenpyroximate was applied at a 1/50 dilution relative to the standard concentration recommended by the manufacturer, the survival rate of Red strains was higher than that of White strains (Tukey’s HSD test; α = 0.05, Table 3). Similarly, a higher survival rate of Red strains than of White strains was observed when acequinocyl and cyenopyrafen were applied at 1/5 and 1/500 dilutions relative to the standard concentrations, respectively. However, no difference was observed between the survival rates of the Red and White strains when any other concentrations of the above three acaricides or any of the tested concentrations of the other two acaricides (milbemycin and bifenazate) were applied (Table 3).

### The presence of White sub-strains possessing high detoxification activity

**Figure 3:**
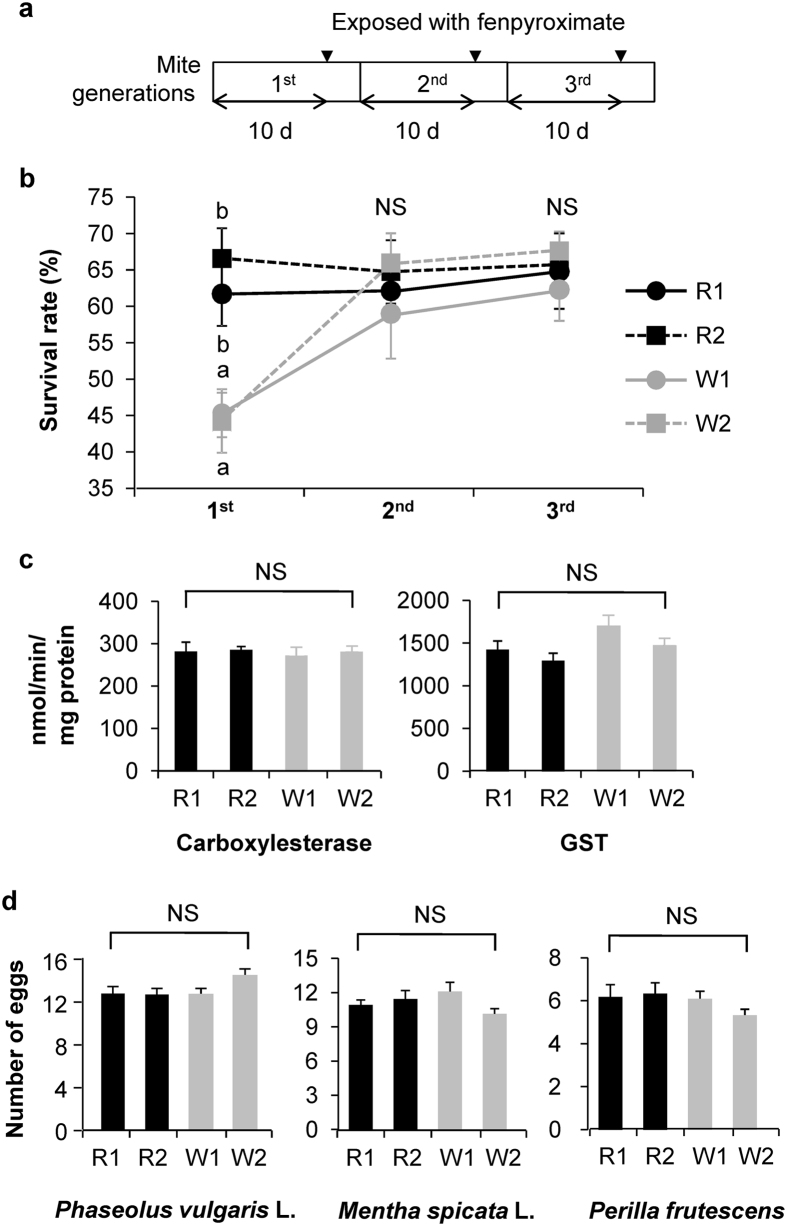
Detoxification activity in the successive generations of Red strains and White strains that survived fenpyroximate treatment. (**a**) Schematic drawing of experimental set-up for generation transition assays. Adults of each original strain (1^st^ generation) were treated with x1/50 fenpyroximate. The individuals that survived were reared to produce the 2^nd^ generation, which was also treated with x1/50 fenpyroximate. This procedure was repeated to produce the 3^rd^ generation. (**b**) Survivability of adult females after exposure to x1/50 fenpyroximate in the 1^st^, 2^nd^ and 3^rd^ generations. Data are shown as the mean survival rate (%) ± standard error (*n* = 10–17), and different small letters indicate that values are significantly different. The data were, after arcsine transformation, subjected to a one-way ANOVA, followed by Tukey’s HSD test (α = 0.05). NS, not significant. (**c**) Detoxification enzyme activity of adult females of the 2^nd^ generation. Data are shown as the mean + standard error (*n* = 4–11). Means were not significantly different (NS; P > 0.05), based on a one-way ANOVA. (**d**) The oviposition rate of the 2^nd^ generation. The number of eggs produced by adult females of the 2^nd^ generation during 2 days on *Phaseolus vulgaris* and Laminaceae species as host was counted. Data are shown as the mean and standard errors (*n* = 17–34). Means were not significantly different (NS; *P* > 0.05), based on a one-way ANOVA.

It should be noted that, in our acaricide resistance assays for White strains at the concentrations noted above, approximately 1/3 to 1/2 of the individual mites could survive. For example, exposure of the White strains to 1/50 fenpyroximate resulted in 38% survivability (W1) and 47% survivability (W2) (see Table 3). The presence of such surviving individuals might be due to either i) variability of exposure efficacy in the bioassay, or ii) the presence of susceptible mites and resistant mites in the White strain at the given acaricide concentrations. In order to test these possibilities, we assessed the acaricide resistance activity in the 2^nd^ and 3^rd^ generations produced as offspring of individual mites that had survived acaricide exposure in the previous generation (Fig. 3).

**Figure 4:**
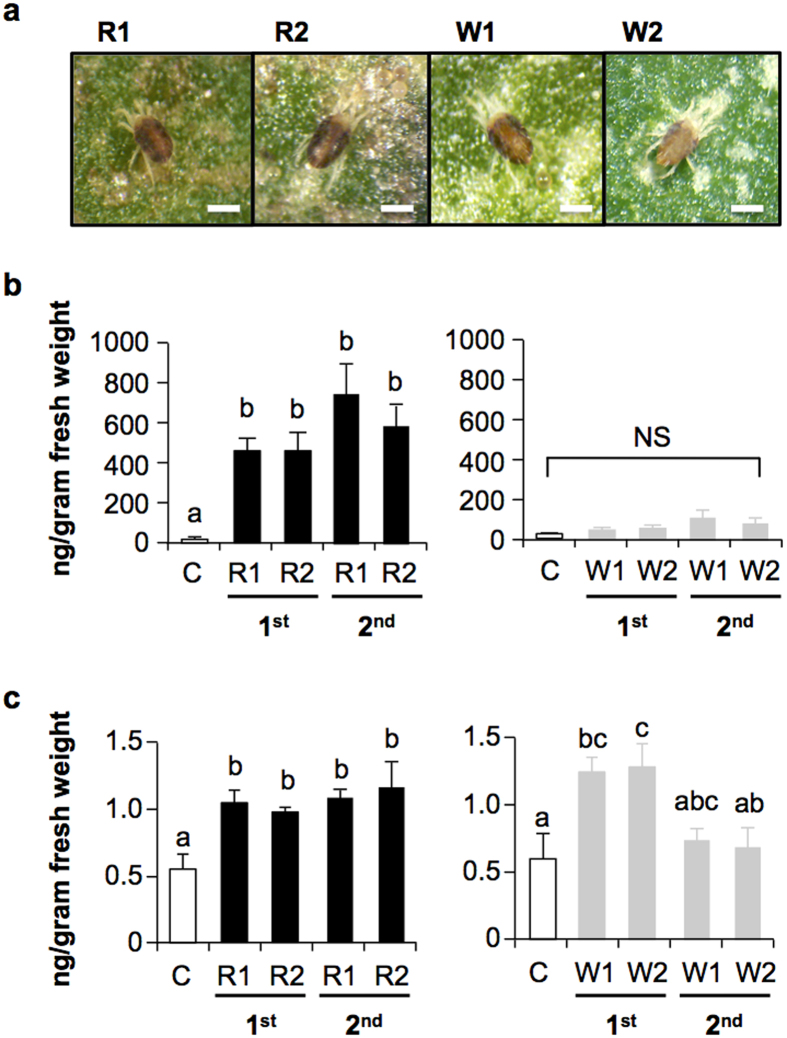
Influence of the 2^nd^ generation of Red strains and White strains that had survived x1/50 fenpyroximate treatment on *Phaseolus vulgaris* leaf defense responses. (**a**) *P. vulgaris* leaves damaged by the 2^nd^ generation. Scale bars = 0.2 mm. Salicylic acid (**b**) and jasmonic acid (**c**) levels in leaves of uninfested *P. vulgaris* plants (control: C) and plants infested with adult females of the original strains (1^st^ generation) or their 2^nd^ generation for 72 h. Data are shown as the mean and standard errors (*n* = 4–8). Means indicated by different small letters are significantly different, based on a Tukey’s HSD test (α = 0.05) after a one-way ANOVA. NS, not significant.

In this assay, Red and White strains that had survived 1/50 fenpyroximate treatment were successively reared until the 3^rd^ generation with 1/50 fenpyroximate treatment in each generation (Fig. 3a). In contrast to the significantly different survival rates between the original Red and White strains (1^st^ generation), the survival rate of the White strains successively reared with 1/50 fenpyroximate treatment was similar to that of the Red strains in the 2^nd^ and 3^rd^ generations (Fig. 3b). The same held when the 2^nd^ generation was exposed to two other acaricides (1/5 acequinocyl or 1/500 cyenopyrafen) (Supplemental Fig. 4). These data indicate that the surviving individuals of both the Red and White strains exhibit high detoxification activity. This was reflected by the similar levels of detoxification enzyme activities (Fig. 3c) and Laminaceae host adaptation (Fig. 3d) between the 2^nd^ generation of Red and White strains. On the other hand, the successive generations of Red and White strains reared with acaricides continued to produce red and white scars (Fig. 4a) and to show similar reproduction (Fig. 3d) on *P. vulgaris* leaves, respectively, like their respective original strains (Supplemental Fig. 1 and Fig. 2).

We next assessed a characteristic feature of these strains by analyzing the levels of accumulation of defense-associated phytohormones (SA and JA) they induced in *P. vulgaris* leaves. It was shown previously that the levels of both SA and JA were increased in *P. lunatus* leaves when damaged by Red strains, while only JA levels were increased when the leaves were damaged by White strains^4^. Likewise, in the current study, increased SA levels were observed in *P. vulgaris* leaves damaged by the original Red strains and their 2^nd^ generation strains compared to the level in uninfested leaves (Tukey’s HSD test; α = 0.05) (Fig. 4b). The SA level was, however, not increased in leaves damaged by either the original White strains or their 2^nd^ generation strains that had survived exposure to acaricide when compared with the level in uninfested leaves.

Increased levels of JA were observed in the leaves damaged by the original Red strains and their 2^nd^ generation strains that had survived exposure to acaricide compared to the level in uninfested leaves (Tukey’s HSD test; α = 0.05) (Fig. 4c). In contrast, the JA levels were increased in leaves damaged by the original White strains but not in leaves damaged by their 2^nd^ generation strains that had survived exposure to acaricide (Fig. 4c).

## Discussion

**Figure 5:**
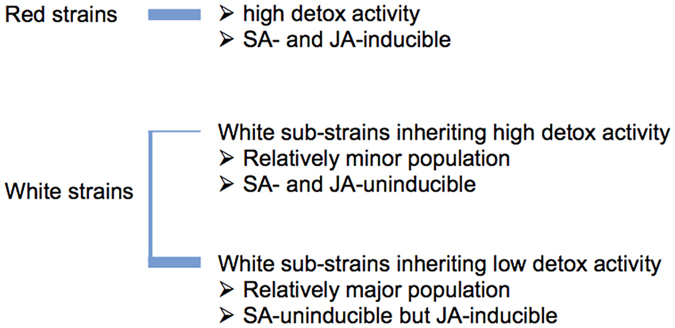
Sub-classified phenotypes of Red and White strains that appeared under environmental pressures.

Phenotypic variations of Tetranychidae are frequently influenced by host plants and environmental conditions, resulting in different phenotypes of reproduction, development, survival, behavior, host adaptation and acaricide resistance^11^,^12^,^13^,^14^. A genome-wide association study of *T. urticae* showed that 24% of all mite genes are differentially expressed upon host plant transfer from *P. vulgaris* to less favorable host plants (i.e., tomato or *Arabidopsis thaliana*), with the most profound changes in genes in the detoxification system (CYPs, carboxyl/cholinesterases, and GSTs)^14^. In the current study, we showed differences of the abilities of detoxification systems between Red and White strains of *T. kanzawai* and, moreover, differences of the detoxification system ability among individual mites within a White strain. This suggested a possible correlation between detoxification systems and host adaptation or acaricide resistance between the two genotypes as well as among their sub-strains (Fig. 5).

Notably, it appeared that the detoxification genes expressed more highly in Red strains are not clustered but rather scattered among several chromosome loci, according to the *T. urticae* genome index (see Tetranychus database IDs in Table 1). This finding suggests the hypothesis that specific master regulator(s) (e.g., transcription factor(s) and/or protein modification enzyme(s)), linked genetically to Red and White scar phenotypes, are involved in regulating functional detoxification enzyme genes and other genes expressed differentially between Red and White strains (Supplemental Table 3). The inheritance of these regulator genes may be closely linked with that of gene(s) responsible for the Red/White scar phenotype. Alternatively, only a limited number of detoxification genes located in a gene cluster(s) genetically linked with regulatory genes for the Red/White scar phenotype might be mostly responsible for the overall detoxification activity in mites.

HSP genes expressed highly in the Red strains might be also responsible for different detoxification activity levels (Table 2). HSPs can be induced by various environmental factors, including pesticides^15^,^16^,^17^, presumably contributing to cellular homeostasis and immunity.

Acaricide resistance traits of spider mites are related to either reduced target site sensitivity resulting from genetic point mutations, or to metabolism of the acaricide before it reaches the target site as a result of transcriptional up-regulation of genes involved in the detoxification process^18^. Various detoxification genes, including genes for CYP, GST, carboxylesterase and ABC transporter, were highly expressed in Red strains (Table 1), resulting in higher enzyme activities of at least GST and carboxylesterase (Fig. 1), in comparison to these genes/enzymes in the original White strains, but not in comparison to minor populations of White sub-strains that inherited high detoxification activity (Fig. 2c). In addition to genome-wide association studies and transcriptome studies^14^,^19^,^20^, functional analysis of detoxification enzymes aiming to elucidate their substrate selectivity and specificity, as well as relevant bioassays, will be essential for elucidating the detailed mechanisms of detoxification of selective chemicals in spider mites.

In comparison to White strains, Red strains were also more able to adapt to two medicinal plant species of Laminaceae (Fig. 2) in which defensive products such as terpenoids are locally and plentifully accumulated in the leaf glandular trichomes^21^,^22^. The relatively high detoxification ability of Red strains would make it easy for them to adapt to such defensive products of Laminaceae. In White strains, it also appeared that at least two sub-strains coexisted in the same population: one possessed strong detoxification activity (high detox White sub-strain), while the other did not (low detox White sub-strain). As a result, the original White strains and high detox White sub-strains showed different host adaptation abilities toward at least two unsuitable host Laminaceae plants (Figs 2 and 3d). These relationships accord with those found for the other trait examined here, i.e., the resistance phenotype toward acaricides, indicating that variations of the detoxification properties among genotypes are closely linked to a broad range of modes of environmental adaption of mites.

However, such a superior ability of host adaptation of Red strains and high detox White sub-strains was not detected in the cases of other host species (*B. rapa* [Brassicaceae], *H. macrophylla* [Hydrangeaceae] or *O. basilicum* [Laminaceae]) examined in the current study (Fig. 2) or in a previous report (*Boehmeria nivea* [Urticaceae], *Pueraria lobata* [Leguminosae], *Cayearatia japonica* [Vitaceae], *Orixa japonica* [Rutaceae], *Nerium indicum* [Loganiaceae], *Rumex crispus* [Polygonaceae], *Erigeron annuus* [Compositae], and *Camellia sinensis* [Theaceae])^3^. Likewise, differences of the survival rates between the Red and White strains were observed only when certain concentrations of acaricides were applied (e.g., 1/50 fenpyroximate) (Table 3). Altogether, these findings suggest that intraspecific *T. kanzawai* genotypes that invest heavily in detoxification power may be advantageous in certain narrow ranges of circumstances in which mites do not face either a very harsh environment (e.g., one in which they suffer exposure to strong acaricides) or a non-favorable environment (e.g., a toxic host habitat).

Considering all these facts, the emergence of Red sub-strains inheriting low detoxification activity remains hard to explain: these sub-strains may occur only rarely, because almost all the Red strain individuals were able to survive when moderate concentrations of acaricides were applied (assuming the survival rate (76%) defined as the basal threshold for survivability in this system; see Table 3). Further ecological and toxicological studies will be required to gain insight into the relationship between the detoxification ability and modes of environmental adaption.

Endogenous SA levels were predominantly increased in *P. vulgaris* leaves when infested by Red strains, but not the original White strains or high detox White sub-strains (Fig. 4b). SA signaling is well known to play a role in the defense response to spider mite damage^23^,^24^,^25^,^26^. However, it remains uncertain whether the low threshold of SA signaling induction in leaves damaged by White strains is advantageous for the mites, because the reproductive performances of the Red and White strains did not differ on the host *P. vulgaris* leaves (Fig. 2). Instead, the higher SA levels in leaves damaged by Red strains may effectively promote indirect plant defenses by increasing the emission of volatile homoterpenes ((*E*)-4,8-dimethyl-1,3,7-nonatriene and (*E,E*)-4,8,12-trimethyl-1,3,7,11-tridecatetraene), as reported in the case of lima bean^4^. As described above, these homoterpenes are infochemicals that act to attract carnivorous natural enemies of spider mites^5^,^6^. Their biosynthesis has been shown to be induced via SA signaling concomitantly with JA signaling in a legume^25^.

In contrast, JA levels were increased in *P. vulgaris* leaves damaged by Red strains and the original White strains, but not by the high detox White sub-strains (Fig. 4c). The presence of individuals that down-regulate JA and/or SA defense signaling pathways improves resource availability for other con- and hetero-specific individuals^23^,^27^,^28^,^29^.

Finally, it should be emphasized that a difference of detoxification activity based on the genotypic difference was observed here only over a narrow range of doses of acaricide and in particular host plants. This suggests that the strong activity of the detoxification system in Red strains would not generally be essential for *T. kanzawai*. In specific cases, such as when the mites are forced to live on unsuitable plant species such as Laminaceae or when they suffer exposure to toxic chemicals (e.g., fenpyroximate), the stronger detoxification system in the Red strains would provide an intraspecific potential for increased fitness.

## Materials and Methods

### Plants

Kidney bean plants (*Phaseolus vulgaris* cv. Nagauzuramame) were grown in soil for 2 weeks. Similarly, *Brassica rapa* var. perviridis, *Mentha spicata* L., *Perilla frutescens* var. crispa ‘chirimen ao jiso’, and *Ocimum basilicum* were grown in soil for 1 month. Individual plants were grown in plastic pots in a climate-controlled room at 25 °C with a photoperiod of 16 h (80 μE m^−2^ s^−1^). Fresh leaves of *Hydrangea macrophylla* (Thunb.) Ser. *f. macrophylla* were collected on the campus of Tokyo University of Science in June 2015.

### Spider mites

*T. kanzawai* mites (Acari: Tetranychidae) were collected from hydrangea in Ami, Ibaraki (360°1′N, 140°12′E) in April, 2004. Collected mites were transferred onto detached *P. vulgaris* leaf discs (25 cm^2^ each), and the discs were placed onto water-saturated cotton in Petri dishes (90 mm diam, 14 mm deep). The dishes were placed in transparent plastic containers under controlled conditions at 25 °C with a photoperiod of 16 h. Small leaf discs (1 cm^2^ each), which were inhabited by ~20 mites and eggs, were collected from the original discs and transferred to fresh leaf discs every 2 weeks for incubation. Fertilized females (and males in the case of the survivability transition assays, see below) 10 days after oviposition were used for experiments.

### Bi-directional isolation of mite strains

To obtain pure mite strains, we screened individual mites based on the color of the scars they made on the surface of leaves, following the method of Matsushima *et al*.^4^. One hundred adult females were randomly isolated from the base population, and the females were individually incubated on *P. vulgaris* leaf discs (1 cm^2^ each) on water-saturated cotton in Petri dishes. After 3–5 days, the 10 females that produced the most distinctive red or white scars were selected and transferred onto a fresh leaf disc and incubated in a single colony. After the populations reached >100 females, the females were individually incubated on leaf discs again, in order to carry out the same process of selection as described above. These selection processes were repeated for more than five generations, thereby resulting in the establishment of a set of independent Red (R1) and White (W1) strains. We repeated the same procedure to establish an additional set of independent Red (R2) and White (W2) strains.

### Measurement of enzyme activity

We determined carboxylesterase activity using 1-naphthyl acetate as substrate in adult female mite homogenates according to the method of Stumpf and Nauen^30^. GST activity was similarly determined using 1-chloro-2,4-dinitrobenzene (CDNB) and reduced glutathione as substrate^30^. The specific carboxylesterase and GST activities were expressed as nmol naphthol/min/mg protein and nmol CDNB conjugated/min/mg protein, respectively. The total protein content in the homogenates was measured according to the method described by Bradford^31^.

### RNA isolation and sequencing

Total RNA from *T. kanzawai* (250–500 females for each sample) was isolated using a Qiagen RNeasy Mini Kit and an RNase-Free DNase Set (Qiagen, Hilden, Germany) following the manufacturer’s protocol and purified to the following approved sample conditions: RNA concentration of 250 ng/μl, RIN (RNA integrity number) of >6.5, and 28S/18S of >1.0. Poly (A)-containing mRNA molecules were purified from total RNA (about 40 μg) using poly-T oligo-attached magnetic beads. Following purification, the mRNA was fragmented into small pieces using divalent cations at elevated temperature.

Illumina libraries from the above-described fragmented RNA (~200 bp) were prepared at the core sequencing facilities at the Beijing Genomics Institute (BGI)-Shenzhen, Shenzhen, China (http://www.genomics.cn). Sequence analysis was performed using the HiSeq 2000 system, with pair-end (2 × 90-bp) reads. Raw sequence data were generated by the Illumina pipeline, and clean reads were generated by filtering out adaptor-only reads, reads containing more than 5% unknown nucleotides, and low-quality reads (reads containing more than 50% bases with Q-value of <20). Only clean reads were used in the following analysis. The sequences from the Illumina sequencing were deposited in DDBJ (accession number: DRA004317).

### *de novo* Transcriptome assembly

RNA-Seq reads were assembled using SOAPdenovo by the program Trinity^32^. The longest assembled sequences are called contigs. Assembly was carried out using default parameters. As a post-processing step, the resultant contigs were further joined into scaffolds by mapping them back to contigs with the paired-end reads. Finally, paired-end reads were used again for gap filling of scaffolds, then gap-filled scaffolds were clustered to remove redundant sequences using the TGICL tool^33^, and overlapped scaffolds were further assembled using Phrap assembly (http://www.phrap.org/) to obtain sequences that had the least Ns and could not be extended on either end. Such sequences are defined as unigenes. The following parameters were used to ensure the quality of the assembly: a minimum of 95% identity between contigs, a minimum of 35 overlapping bases, a minimum quality score of 35 and a maximum of 20 unmatched overhanging bases at sequence ends. The identity of the resulting assemblies was verified and checked for contamination through BLASTN searches against a custom database of 18S ribosomal RNA sequences (E-value < 1e-10)^34^.

Unigene/contig expression levels were calculated using the ERANGE package. The formula was FPKM (Fragments Per Kilobase of exon per Million mapped fragments = (1000000 * C)/(N * L * 1000)), where FPKM(A) is the expression of gene A, C is the number of fragments that uniquely aligned to gene A, N is the total number of fragments that uniquely aligned to all genes, and L is the number of bases in gene A. The calculated gene expression (FPKM) was directly used for comparing the difference of gene expression level among samples.

We used an algorithm developed originally by BGI to identify differentially expressed genes between two samples. We denoted x as the number of fragments that could uniquely map to gene A. For each transcript representing a small fraction of the library, *p(x*) closely followed the Poisson distribution^35^. The total fragments number of sample 1 is N1, and the total fragments number of sample 2 is N2; gene A has x fragments in sample 1 and y fragments in sample 2. The probability that gene A is expressed equally between two samples was calculated with the following formula: *p(i*|*x*) = (*N*_2_/*N*_1_)^*i*^ (*x* + *i*)!/*x*!*i*!(1 + *N*_2_/*N*_1_)^(*x*+*i*+1)^. We used data with False Discovery Rate (FDR^36^) ≤0.001 and a ratio larger than 2. We also used data showing no expression (FPKM: 0) in either Red or White strains. Since we assessed only one biological replication for each strain, note that the above *P* value might be inaccurate, and therefore we relied on these data just for screening of the candidate genes expressed differentially between Red and White strains.

To annotate the dataset, the unigenes were searched for sequence similarity using BLASTX^34^ against the NR, Swiss-Prot, KEGG, COG and GO databases and the reported *T. urticae* genome sequences (https://bioinformatics.psb.ugent.be/gdb/tetranychus/), and then the best-hit for each search was retrieved and annotated to each unigene/contig (E-value < 1e-5). The results from the similarity searches with the Swiss-Prot and *T. urticae* genome sequences are shown in Supplemental Table 2.

### RT-qPCR

Approximately 100 mites were homogenized in liquid nitrogen, and total RNA was isolated and purified as described above. First-strand cDNA was synthesized using ReverTra Ace qPCR RT Master Mix with gDNA Remover (Toyobo, Osaka, Japan) and 0.5 μg of total RNA first at 37 °C for 5 min for the DNase reaction and second at 37 °C for 15 min for the RT reaction. Real-time PCR was performed using a CFX Connect Real-Time PCR detection system (Bio-Rad, Hercules, CA, USA) using THUNDERBIRD SYBR qPCR Mix (Toyobo) and gene-specific primers (Supplemental Table 4). The following protocol was used: initial polymerase activation: 60 s at 95 °C; 40 cycles of 15 s at 95 °C and 30 s at 60 °C; and then melting curve analysis preset by the instrument. Relative transcript abundances were determined after normalization of raw signals with housekeeping transcript abundance of a histone H3 gene (XM_015926956)^37^. Replicate analyses were conducted with five or six independent samples.

### Oviposition assays

A *T. kanzawai* adult female (10 days old after oviposition) was transferred onto a leaf disc (1.8 cm^2^) of *P. vulgaris* or other plant host on wet cotton in a plastic Petri dish. Each dish containing 10 discs was incubated in a climate-controlled room at 25 °C with a photoperiod of 16 h, and the number of eggs laid by each female was counted after 48 h.

### Acaricide resistance assays

We prepared aqueous solutions of Starmite^®^ (cyenopyrafen, Nissan Chemical Industries, Ltd., Tokyo, Japan), Danitoron^®^ (fenpyroximate, Nihon Nohyaku Co., Ltd., Tokyo, Japan), Maitokone^®^ (bifenazate, Nissan Chemical Industries, Ltd., Tokyo, Japan), Koromaito^®^ (milbemycin, Mitsui Chemicals Agro, Ltd., Tokyo, Japan) and Kanemaito^®^ (acequinocyl, Agro-Kanesho Co., Ltd., Tokyo, Japan), at x1, x1/5, x1/50 and x1/500-dilutions relative to the standard concentrations recommended by manufacturers. Ten adult females (10 days old) after the oviposition were transferred onto a *P. vulgaris* small leaf disc (1.8 cm^2^) on wet cotton in a plastic Petri dish (90 mm dia.). Each dish contained 10 discs. Following incubation at 25 °C for 24 h with a photoperiod of 16 h, the discs were dipped in the acaricide solutions (5 ml) for 10 s, and air-dried for 5 min. Survival rate of mites on the disc was determined after 24 h. Individual mites were scored as “dead” when they did not respond to brushing. Leaf discs (containing 10 adult females) dipped in water served as a control.

### Generation transition assays

Ten adult females and 20 adult males of each strain after the oviposition were transferred separately onto a *P. vulgaris* small leaf disc (2.3 cm^2^), and the disc was dipped in fenpyroximate solution (x1/50 dilution) for 10 sec (see above for details). All the surviving females and males were transferred onto a fresh leaf disc (25 cm^2^) and then incubated to produce the 2^nd^ generation. Ten adult females and 20 males of this 2^nd^ generation at 10 days after the oviposition were again transferred onto a small leaf disc (1.8 cm^2^), and the disc was dipped in fenpyroximate solution (x1/50 dilution) for 10 sec. Likewise, all the surviving females and males were incubated to produce the 3^rd^ generation. For assays, adult females 10 days after the oviposition were dipped in fenpyroximate solution [x1/50 dilution], acequinocyl [x1/5 dilution] or cyenopyrafen [x1/500 dilution] for 10 sec, and the survival rate of mites was determined after 24 h (see above).

### Determination of JA and SA levels

*P. vulgaris* plants were infested with 40 adult females of each strain for 72 h. We determined leaf JA and SA levels using a liquid chromatography-tandem mass spectrometry (LC/MS/MS) system according to Ozawa *et al*.^38^ with minor modifications. Frozen leaves (0.5 g) ground in liquid nitrogen were homogenized with ethyl acetate (2.5 ml), spiked with 10 ng of d_2_-JA (Tokyo Chemical Industries Co., Ltd., Tokyo, Japan) and 1 ng of d_4_-SA (C/D/N Isotopes Inc., Pointe-Claire, QC, Canada) as internal standards. After centrifugation of the mixture at 5000 rpm for 10 min at 4 °C, 1 ml of supernatant was transferred to a 1.5-mL tube and then evaporated to dryness under vacuum. The residue was suspended in 50 μl of 70% methanol/water (v/v) and centrifuged to clarify the liquid phase, and the supernatant was analyzed using an LC/MS/MS system (LCMS-8040, Shimadzu Co. Kyoto, Japan). Separation by HPLC was performed with a Mightysil RP-18 GP column (100 × 2.0 mm, 3 μm particle size, Kanto Chemical, Tokyo, Japan) at a flow rate of 200 μl min^−1^. A linear gradient [0.1% formic acid aq. (A) and methanol (B), 5–95% B/(A + B) for 16 min] was applied. Concentrations of JA, d_2_-JA, SA and d_4_-SA were determined by multiple reaction monitoring (MRM). The monitored mass transitions were *m/z* 209 to *m/z* 59 for JA, *m/z* 211 to *m/z* 59 for d_2_-JA, *m/z* 136 to *m/z* 93 for SA and *m/z* 141 to *m/z* 97 for d_4_-SA. The conditions for MS were optimized for MRM using authentic compounds of d_2_-JA (Tokyo Chemical Industries, Tokyo, Japan), d_4_-SA (C/D/N Isotopes), JA (Tokyo Chemical Industries) and SA (Wako Pure Chemical Industries, Ltd, Osaka, Japan).

### Statistics

For multiple comparisons of data of the *P. vulgaris* leaf JA/SA levels as well as the number of eggs and the enzyme activities, data were analyzed by a one-way ANOVA, followed by Tukey’s HSD test. To compare data of the acaricide resistance assays, the original percentage data were subjected to a one-way ANOVA after arcsine transformation, followed by Tukey’s HSD test. In all cases, the level of significance was set to α = 0.05.

## Additional Information

**How to cite this article:** Ozawa, R. *et al*. Intraspecific variation among Tetranychid mites for ability to detoxify and to induce plant defenses. *Sci. Rep.* **7**, 43200; doi: 10.1038/srep43200 (2017).

**Publisher's note:** Springer Nature remains neutral with regard to jurisdictional claims in published maps and institutional affiliations.

## Supplementary Material

Supplemental Table 1

Supplemental Table 2

Supplemental Table 3

Supplemental Table 4

Supplemental Figures

## Figures and Tables

**Table 1:** Detoxification genes differentially expressed between Red and White strains.

	Gene ID	Tetranychus database ID	FPKM				Fold expression^a^			
			R1	R2	W1	W2	R1/W1	R2/W1	R1/W2	R2/W2
Cytochrome P450	CL86.Contig9	tetur27g00340	1.18	1.4	0	0	—	—	—	—
	CL86.Contig15	tetur27g00340	3.27	3.54	0.73	1.08	2.16	2.28	1.60	1.72
	CL1948.Contig1	tetur36g00920	4.59	4.28	1.42	1.40	1.69	1.59	1.71	1.61
	Unigene8610	tetur03g05010	3.54	4.64	1.13	1.05	1.65	2.04	1.75	2.14
	Unigene10928	tetur07g06460	3.62	6.95	0.34	0.88	3.41	4.35	2.04	2.98
	Unigene12355	tetur11g05000	1.02	1.39	0.40	0.36	1.36	1.82	1.50	1.95
Glutathione *S*-transferase	CL1160.Contig1	tetur03g07920	32.77	36.02	6.83	7.23	2.26	2.40	2.18	2.32
	CL1160.Contig2	tetur26g02802	16.83	29.62	3.30	3.00	2.35	3.17	2.49	3.30
	CL1160.Contig4	tetur26g01450	15.26	21.88	4.97	2.99	1.62	2.14	2.35	2.87
	CL2286.Contig8	tetur29g00220	9.19	13.57	2.67	1.20	1.78	2.35	2.93	3.50
	Unigene4639	tetur26g01460	5.31	7.05	0.73	0.34	2.87	3.28	3.96	4.37
	Unigene4640	tetur03g07920	5.25	6.92	0.62	0.38	3.09	3.48	3.78	4.18
	Unigene4642	tetur03g07920	3.63	6.60	0.37	0.25	3.29	4.15	3.83	4.70
	Unigene4643	tetur03g07920	3.90	6.59	0.43	0.32	3.17	3.93	3.62	4.37
Carboxylesterase	CL187.Contig8	tetur11g01570	0.08	0.59	0	0	—	—	—	—
	Unigene1530	tetur11g01570	0.50	3.25	0	0.02	—	—	4.46	7.16
	Unigene1585	tetur11g01570	0.36	1.87	0	0.01	—	—	5.92	8.31
	Unigene1594	tetur11g01570	0.08	0.67	0	0.01	—	—	3.14	6.12
	Unigene1597	tetur11g01570	0.13	0.72	0	0.01	—	—	4.52	6.96
	Unigene1626	tetur11g01570	0.09	0.68	0	0.01	—	—	3.13	6.11
	CL1124.Contig1	tetur35g00210	11.44	12.23	4.08	3.99	1.49	1.59	1.52	1.62
	CL1837.Contig2	tetur01g08680	23.37	5.87	1.36	0.94	4.10	2.11	4.64	2.65
ABC transporter	CL210.Contig1	tetur40g00010	51.20	43.98	20.79	16.27	1.30	1.08	1.65	1.43
	CL210.Contig5	tetur03g09800	6.37	4.57	1.41	1.01	2.17	1.69	2.65	2.17
	CL210.Contig11	tetur03g09800	4.14	4.52	1.25	1.24	1.73	1.85	1.73	1.86
	CL210.Contig17	tetur40g00010	7.18	5.47	1.69	1.30	2.09	1.70	2.47	2.07
	CL210.Contig18	tetur40g00010	7.14	5.06	1.66	1.17	2.11	1.61	2.61	2.11
	CL1338.Contig2	tetur32g01330	6.06	6.62	2.45	1.63	1.31	1.43	1.90	2.02
	CL1587.Contig1	tetur09g04610	8.90	11.49	3.80	3.17	1.23	1.60	1.49	1.86
	CL2055.Contig1	tetur11g04030	1.00	1.06	0.25	0.20	2.00	2.08	2.33	2.40
	CL2870.Contig2	tetur20g02610	6.13	7.85	2.87	2.55	1.10	1.45	1.27	1.62
	CL4679.Contig2	tetur32g00490	22.32	28.06	10.50	10.49	1.09	1.42	1.09	1.42
	Unigene7691	tetur04g07910	1.40	3.04	0.55	0.28	1.35	2.47	2.31	3.43

FPKM, Fragments Per Kilobase of exon per Million mapped fragments.

^a^Fold change is log2(R1 or R2/W1 or W2), >log2 = 1 or <log2 = −1.

**Table 2:** Heat shock protein genes differentially expressed between Red and White strains.

	Gene ID	Tetranychus database ID	FPKM				Fold expression^a^			
			R1	R2	W1	W2	R1/W1	R2/W1	R1/W2	R2/W2
DnaJ	CL1638.Contig1	tetur01g13410	32.36	36.63	7.41	6.48	2.13	2.31	2.32	2.50
DnaJ	CL1638.Contig2	tetur01g13410	9.93	8.46	0.67	0.56	3.90	3.67	4.15	3.92
DnaJ	CL1638.Contig4	tetur01g13400	90.78	80.10	34.52	26.66	1.39	1.21	1.77	1.59
DnaJ	CL2475.Contig2	tetur08g07280	10.06	6.72	0.99	1.45	3.34	2.76	2.80	2.22
DnaJ	CL4208.Contig1	tetur15g02000	10.69	21.12	2.67	1.85	2.00	2.99	2.53	3.51
DnaJ	Unigene11337	tetur13g02530	10.04	12.38	4.72	4.05	1.09	1.39	1.31	1.61
DnaJ	Unigene14563	tetur97g00050	434.42	1758.76	46.20	113.91	3.23	5.25	1.93	3.95
Hsp27	Unigene9853	tetur22g01530	57.63	87.84	9.55	9.72	2.59	3.20	2.57	3.18
Hsp60	CL4306.Contig1	tetur02g03850	12.89	13.24	3.59	3.36	1.84	1.88	1.94	1.98
Hsp60	CL4306.Contig2	tetur02g03850	9.72	8.03	2.99	2.78	1.70	1.43	1.81	1.53

FPKM, Fragments Per Kilobase of exon per Million mapped fragments.

^a^Fold change is log2(R1 or R2/W1 or W2), >log2 = 1 or <log2 = −1.

**Table 3:** Survival rate of Red and White strains exposed to acaricides.

Trade name (Common name)	Recommended concentration (ppm)	Surival rate (%)											
		x1/5^a^				x1/50^a^				x1/500^a^			
		R1	R2	W1	W2	R1	R2	W1	W2	R1	R2	W1	W2
Danitoron (Fenpyroximate)	50	1	12	9	6	**73**	**72**	**38**	**47**	90	82	81	89
Kanemaito (Acequinocyl)	150	**67**	**70**	**40**	**48**	91	82	86	77	79	90	86	84
Koromaito (Milbemycin)	10	0	0	0	0	0	0	0	0	32	34	31	33
Maitokone (Bifenazate)	200	0	0	0	0	0	1	0	0	70	51	54	59
Starmite (Cyenopyrafen)	150	0	0	0	0	0	0	0	2	**82**	**83**	**47**	**57**

Data are shown as the mean survival rate (%) of adult females on a leaf disc exposed to each acaricide (*n* = 5–39). Data sets with the bold letters are significantly different between Red and White strains, based on, after arcsine transformation, a one-way ANOVA, followed by Tukey’s HSD test. (α = 0.05). Both Red and White strains dipped with water alone served as control and resulted in about 76% survival rate: this value was defined as basal threshold for survivability in this system.

^a^Dilution ratio from the standard concentrations recommended by manufacturers.
