# Supplementary material for: Intraspecific variation among Tetranychid mites for ability to detoxify and to induce plant defenses
Source: Sci Rep. 2017 Feb 27;7:43200. doi: 10.1038/srep43200 (PMC5327432; doi:10.1038/srep43200)
Supplement: Supplemental Figures [file srep43200-s5.docx]

**Intraspecific variation among Tetranychid mites for ability to detoxify and to induce plant defenses**

Rika Ozawa^1^, Hiroki Endo^2^, Mei Iijima^2^, Koichi Sugimoto^1^, Junji Takabayashi^1^, Tetsuo Gotoh^3^ and Gen-ichiro Arimura^2^

^1^Center for Ecological Research, Kyoto University, Otsu 520-2113, Japan. ^2^Department of Biological Science & Technology, Faculty of Industrial Science & Technology, Tokyo University of Science, Tokyo 125-8585, Japan. ^3^Laboratory of Applied Entomology and Zoology, Faculty of Agriculture, Ibaraki University, Ibaraki 300-0393, Japan.

**
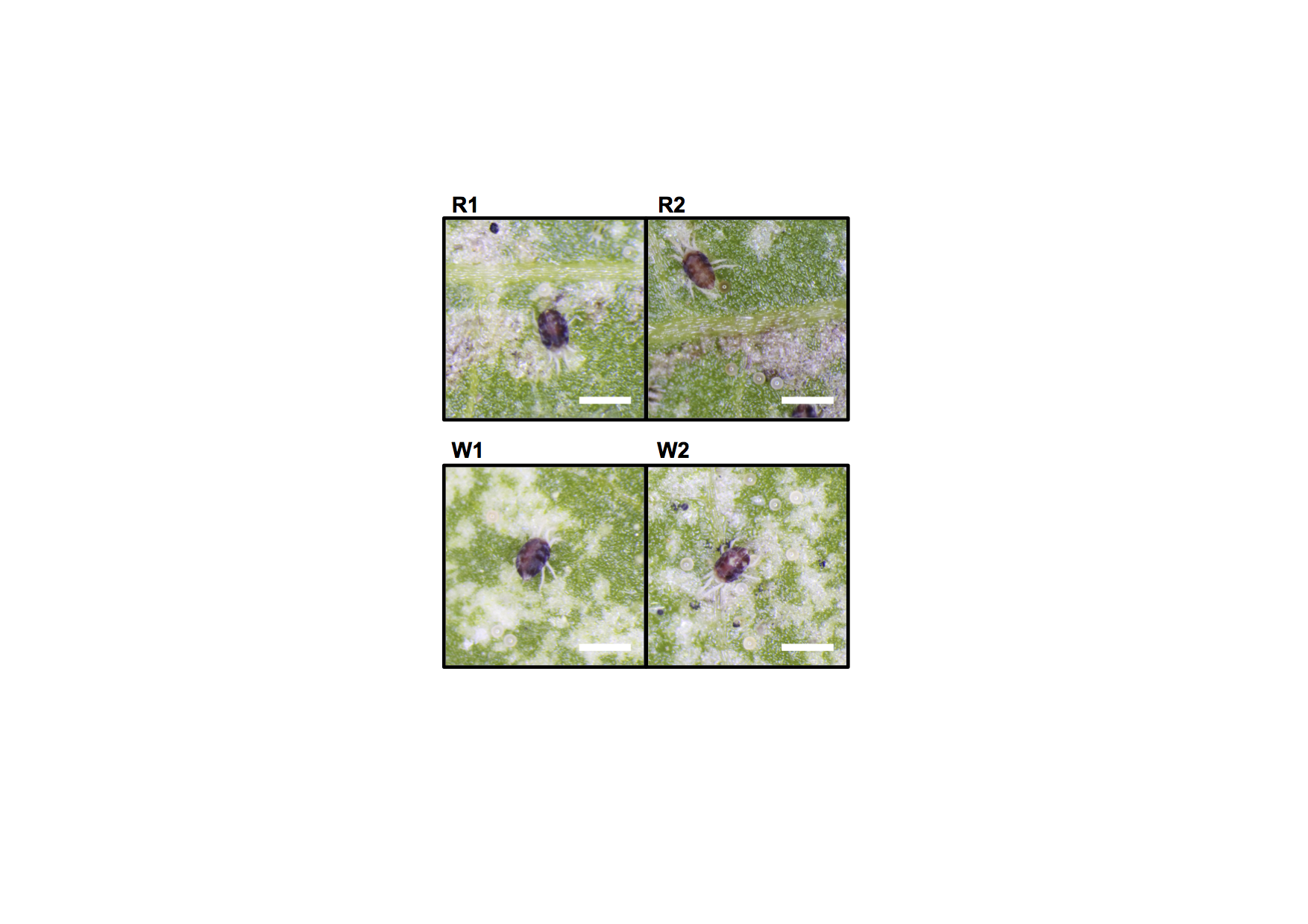
**

**Supplemental Figure 1. *Phaseolus vulgaris* leaves damaged by Red strains (R1 and R2) or White strains (W1 and W2).** Scale bars = 0.5 mm.

**
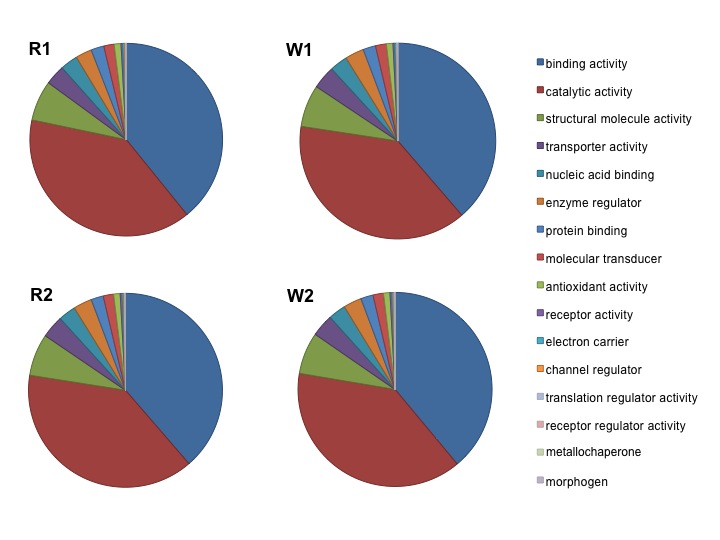
**

**Supplemental Figure 2. Distribution of genes expressed in distinct strains.** The major categories of level 2 molecular functions from a Gene Ontology (GO) analysis are shown (*n* = 10195, 11439, 10874 and 10952 for R1, R2, W1 and W2, respectively).

**Supplemental Figure 3.** Expression levels of representative genes for detoxification enzymes (cytochrome P450 [CYP, CL1948.Contig1], glutathione *S*-transferase [GST, CL1160.Contig2], carboxylesterase [CL1837.Contig2] and ABC transporter [CL210.Contig5]) and heat shock proteins (DnaJ [CL1638.Contig1] and hsp27 [Unigene9853]) in the body of Red strains (R1 and R2) and White strains (W1 and W2). Transcription levels of genes were normalized by those of a histone H3 gene ([tetur05g07250](http://metazoa.ensembl.org/Tetranychus_urticae/Gene/Summary?db=core;g=tetur05g07250;r=HE587308:274000-284000;tl=BlvXYjQnefzFH0TE-10664601-188545868)). Data represent the mean and standard error (*n* = 5-6). Different letters indicate significant differences between treatments (Tukey’s HSD test; α = 0.05) after a one-way ANOVA.

**
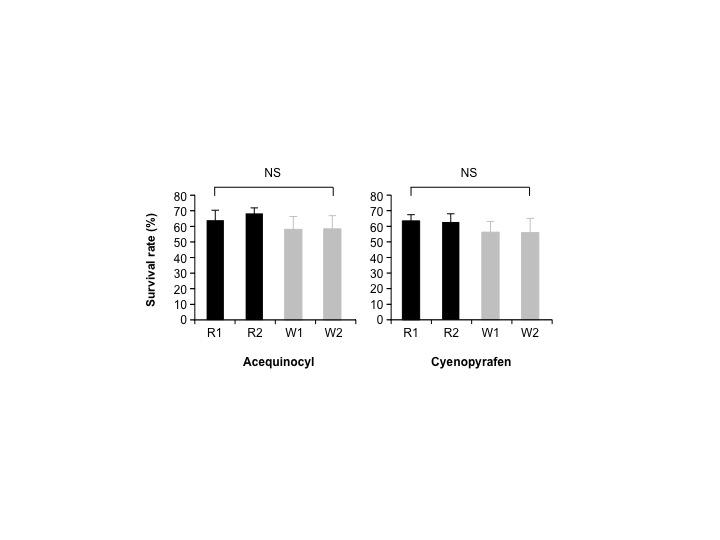
**

**Supplemental Figure 4. The survival rate (%) of the 2^nd^ generation after exposure to either x1/5 acequinocyl or x1/500 cyenopyrafen.** Adult females of the 2^nd^ generation of Red strains (R1 and R2) and White strains (W1 and W2) that had survived x1/50 fenpyroximate treatment were treated with either x1/5 acequinocyl or x1/500 cyenopyrafen. Data are shown as the mean + standard error (*n* = 13-26). NS, not significant (*P* > 0.05; one-way ANOVA after arcsine transformation).
